# Quality changes to approved biotherapeutic product: Simulated case studies on reporting categories & supporting data requirements

**DOI:** 10.1016/j.biologicals.2019.10.008

**Published:** 2019-11

**Authors:** Hugo Hamel, Hye-Na Kang

**Affiliations:** a Health Canada, Tunney's Pasture, A/L 0603C2, Ottawa, Canada; b World Health Organization, Avenue Appia 20, Geneva, Switzerland

**Keywords:** Biotherapeutic product, Post-approval change, Quality change, Case study, Implementation workshop, WHO

## Abstract

Changes are essential for the continual improvement of the manufacturing process and for maintaining state-of-the-art controls on biotherapeutic products and such changes often need to be implemented after the product has been approved. WHO guidelines on procedures and data requirement for changes to approved biotherapeutic products were issued in 2017 to provide guidance to national regulatory authorities and manufacturers on the regulation of changes to already licensed biotherapeutic products in order to assure their continued quality, safety and efficacy, as well as continuity of supply and access. The case studies in this article were prepared to be used for WHO implementation workshops. Using these case studies, an interactive discussion was carried out among the workshop participants, and this article reflects the outcomes of case study exercise and lessons learnt from the 1st implementation workshop on the guidelines held on 25–26 June 2019, Seoul, Korea.

## 1 Introduction

After approval, changes to the manufacturing process of a medicine often occur. Medicines undergo changes during the product life-cycle, and the reasons for such changes include, e.g. improvement of the manufacturing process, increase in scale, a site change, improvement of product stability, or compliance with changes in regulatory requirements [1]. The changes are welcome as these are often improvements, but a small change in manufacturing processes may have a large impact on the final product, and for example lead to serious adverse effects in recipients. For instance, a formulation change of erythropoietin caused enhanced development of pure red cell aplasia [2]. Thus, the manufacturing process changes for licensed medicines, particularly biologicals should be approved by relevant regulators based on a demonstration of product comparability before and after the manufacturing change [1].

In response to requests from its Member States [3,4], WHO developed new guidelines on procedures and data requirement for changes to approved biotherapeutic products (hereafter referred to as 'the Guidelines') which were adopted by the WHO Expert Committee on Biological Standardization (ECBS) in 2017 and published in WHO Technical Report Series No. 1011, Annex 3 [1].

“Prior to implementing a change with a potential impact on quality, the marketing authorization holder should demonstrate through appropriate studies (analytical testing, functional assays and, if needed, clinical and/or nonclinical studies) that the pre-change and post-change products are comparable in terms of quality, safety and efficacy” [1]. The Guidelines provide comprehensive lists of quality changes and the type of information that should be included in a supplement application in Appendix 2 (for the drug substance and intermediates) and in Appendix 3 (for the drug product) [1]. The information provides guidance on: 1) the conditions to be fulfilled to classify a specific change as major, moderate or minor; 2) the specific data requirements to support approval of a proposed change; and 3) the reporting category (major, moderate or minor quality change).

WHO has organized several implementation workshops on various guidelines in order to facilitate the implementation of evaluation principles in WHO guidelines into regulatory and manufacturing practices in countries. The case studies in this article were intentionally developed for the purpose of group work practice at WHO implementation workshops on the Guidelines on procedures and data requirement for changes to approved biotherapeutic products. By exercising the practical use of Appendices 2 and 3 as well as using own judgement for the change which is not listed in the Appendices, it would contribute to better understanding of the evaluation principles outlined in the Guidelines [1].

## 2 Methodology

The case studies were used in the 1^st^ implementation workshop for the Guidelines on 25–26 June 2019, Seoul, Korea. In total, 7 case studies were presented during the workshop, but a sample of 4 case studies are included in this article that are representative of the most common quality changes made by the manufacturers or the marketing authorization holders (MAHs). The information and data in these case studies are fictitious and do not represent products approved or under development. In order to practice the case studies through group discussions, workshop participants including 27 regulators from 21 countries that covered all 6 WHO regions (Brazil, Canada, China, Cuba, Egypt, Ghana, Indonesia, India, Iran, Japan, Jordan, Korea, Malaysia, Peru, Russia, Singapore, Thailand, Ukraine, UK, USA, Zambia) and 26 manufacturers took part in the case study exercise during the workshop. Participants were divided into 7 groups, i.e. 8 persons/group, comprising even distribution between WHO regions and the ratio of regulators vs. manufacturers. The plan for the group-work including the case study description was sent to all participants 2 weeks before the workshop.

## 3 Description of case studies

### 3.1 Case study 1: Change in the quality control testing site for the final product

#### 3.1.1 Rationale for the change

In order to increase the quality control testing capacity of the drug product, the MAH of a monoclonal antibody proposes to add two new facilities for drug product release testing. The tests proposed to be transferred are the following:

• Clarity, Opalescence & Coloration
• Identity test by Peptide mapping
• Purity by IE-HPLC
• Purity testing by CE-SDS
• Purity testing by SEC-HPLC
• Endotoxin
• Sterility
• Osmolality
• Potency (cell-based assay)

#### 3.1.2 Description

The first proposed facility (Site A) is within a good manufacturing practice (GMP)-approved site belonging to the company and the second proposed facility (Site B) is a contract facility not approved in the current marketing application. All above described tests are transferred at both sites. Some are pharmacopoeial; others are non-pharmacopoeial.

#### 3.1.3 Data supporting the change

• Technical transfer data

For the non-pharmacopoeial assays, two samples and the reference standard were tested at both sites and the results compared in order to demonstrate adequate technical transfer. Verification of the pharmacopoeial assays has been performed.

• Evidence of GMP compliance

A Certificate of GMP compliance by the regional regulatory authority is included.

#### 3.1.4 Questions

• What type of category is appropriate for this change? For Site A? For Site B?
• Is the data set appropriate to support approval of the change?

### 3.2 Case study 2: Change to the cell banks

#### 3.2.1 Case study 2-1: Change to the working cell banks (WCBs)

##### 3.2.1.1 Rationale for the change

• Updated qualification protocol for establishing a new WCB
• Addition of a new site for viral safety testing of the WCB

##### 3.2.1.2 Description

• The WCB qualification protocol has been updated to change the cell line authenticity method. Instead of using isoenzyme analysis, the MAH proposes to use a DNA fingerprinting-based assay. This is due to the limited commercial availability of the isoenzyme analysis test and greater sensitivity of the DNA fingerprinting-based assay.
• The new testing site is Company ABC located in Germany and the sponsor confirmed that no changes have been made to the storage conditions used for the cell bank, and that the transport conditions remain within the validated condition.

##### 3.2.1.3 Data supporting the change

• Updated WCB qualification protocol
• Confirmation that Company ABC is a GMP compliant testing laboratory. This location has never been inspected by any EU authorities and therefore does not have a GMP certificate of compliance.

##### 3.2.1.4 Questions

• What type of category is appropriate for these changes?
• Can they be filed together?
• Is the data set appropriate to support approval of the change?

#### 3.2.2 Case study 2-2: Change to the master cell banks (MCBs)

##### 3.2.2.1 Questions

• If a new MCB was being re-established, what would be the reporting category if:
  o The new MCB was generated from the same expression construct with same or closely related cell line (e.g. from CHO–K1 to CHO-DG44)
  o The new MCB was generated from a different expression construct with the same coding sequence and the same cell line
  o The new MCB was generated from a different cell substrate (e.g. from CHO hamster cell line to NS0 murine cell line)

### 3.3 Case study 3: Change to the fermentation process or cell culture

#### 3.3.1 Rationale for the change

• Introduction of Animal Component Free (ACF) media to the drug substance manufacturing process.

#### 3.3.2 Description

• The new media ACF is being implemented to replace the currently used Animal Component Containing media
• As this is animal-free medium, there is no risk of introducing new viruses
• The MAH stated that the impurity profile of the drug substance remains within the approved limits.

#### 3.3.3 Data supporting the change

• Certificate of Analysis for all compendial materials that composed the ACF media. For the non-compendial materials, in-house standards are available
• Validation data are available to support the revised cell culture
• Comparability data of the pre-change product and post-change product
• Batch data from one commercial-scale drug substance batch manufactured with the new cell culture media
• 3 months of stability data at 2–8 °C and 3 months at 25 °C for the post-change batch

#### 3.3.4 Questions

• What type of category is appropriate for this change?
• Is the data set appropriate to support approval of the change?

### 3.4 Case study 4: Change in reference standards

#### 3.4.1 Questions

• If a new reference standard was being generated, what would be the reporting category if:
  • The new reference standard was generated from the approved primary reference standard according to the approved protocol and was used for a physicochemical test at release.
  • The new reference standard was generated from an unapproved primary reference standard.
  • The new reference standard was qualified against the approved reference standard and was used as a control sample for an in-process control test.
  • The new reference standard used for the potency assay test at release was changed from in-house to an international standard.
  • A new lot of reference standard was qualified against the approved reference standard according to a modified qualification protocol (the MAH removed one of the purity test).
  • A new lot of reference standard was qualified against the approved reference standard according to a modified qualification protocol (the MAH tightened the acceptance criteria for one of the purity test).

## 4 Evaluation of the case studies and key discussion points

### 4.1 Case study 1: Change in the quality control testing site for the final product

#### 4.1.1 Reporting categories

For a change involving the quality control testing of the drug product, Change 51 listed in Appendix 3 of the Guidelines should be followed [1]. Accordingly, the requirements are different for the two sites.

**Table:** 

Description of change	Conditions to be fulfilled	Supporting data	Reporting category
51. **Change affecting the quality control testing of the drug product (release and stability), involving the following:**			
Note: Transfer of testing to a different facility within a GMP-compliant site is not considered to be a reportable change but is treated as a minor GMP change and is reviewed during inspections.			
a. Transfer of the quality control testing activities for a non-pharmacopoeial assay (in-house) to a new company not approved in the current marketing authorization or licence or to a different site within the same company	None	1, 2	Moderate
	1–3	1, 2	Minor
b. Transfer of the quality control testing activities for a pharmacopoeial assay to a new company not approved in the current marketing authorization or licence	None	1, 2	Moderate
	1	1, 2	Minor
**Conditions**			
1. The transferred quality control test is not a potency assay or bioassay.			
2. There are no changes to the test method.			
3. The transfer is within a facility approved in the current marketing authorization for the performance of other tests.			
**Supporting data**			
1. Information demonstrating technology transfer qualification for the non-pharmacopoeial assays or verification for the pharmacopoeial assays.			
2. Evidence that the new company/facility is GMP-compliant.			

For Site A:

Transfer of testing to a different facility within a GMP-approved site is not considered to be a reportable change but is treated as a minor GMP change and is reviewed during inspection. Therefore, the transfer of all the methods is considered a ***“Quality change with no impact”***.

For Site B:

Non-pharmacopoeial and pharmacopoeial assays are proposed to be transferred to a new company not approved in the current marketing application.

For the non-pharmacopoeial assays, Conditions 1, 2 and 3 must be met in order to file the change as a Minor change. The third condition (i.e. the transfer is within a facility approved in the current marketing authorization for the performance of other tests) is not met. Therefore, a pre-approval supplement (PAS) categorized as ***Moderate change*** should be filed to support the transfer of the following non-pharmacopoeial assays:

• Peptide mapping
• IE-HPLC
• CE-SDS
• SEC-HPLC
• Potency

For the pharmacopoeial methods (i.e. Clarity, Opalescence & Coloration, Endotoxin, Sterility, Osmolality), as none are considered potency assay, they should be categorized as ***Minor changes*** according to Change 51b [1]. However, as it is encouraged that the MAHs describe the minor changes that are related/consequential to a Moderate or Major change in the submitted PAS [1], the transfer of the pharmacopoeial assays should be described in a ***Moderate change*** filed to support the transfer of the non-pharmacopoeial assays.

#### 4.1.2 Supporting data

For Site A, as the transfer of testing to a different facility within a GMP-approved site is not considered to be a reportable change but is instead treated as a minor GMP change, the supporting data do not need to be submitted to the national regulatory authority (NRA). However, the MAHs should ensure that the methods have been adequately transferred and the supporting data available for review during inspection. For Site B, it was agreed by the participants that what was proposed to be submitted by the MAH to support the technical transfer between the donor site and the receiving site were considered acceptable and were in line with the requirements of Change 51a [1].

#### 4.1.3 Key discussion points

Two groups classified the transfer of testing to Site A as a quality change with no impact whereas one group classified the change as a Minor change and another group as Minor change with no impact. It was clarified that quality changes may be categorized either as a ***Major, Moderate, Minor*** or ***Quality change with no impact***. The reporting category “*Minor change with no impact*” does not exist. Regarding the group that reported the transfer of testing to site A as a Minor Change, it was clarified that according to the examples of changes listed as Quality change with no impact in the Guidelines [1], the transfer of quality control testing activities to a different facility within a GMP-approved site falls in this category.

For the transfer of testing activities to Site B, all group adequately classified the transfer of the non-pharmacopoeial assays as a ***Moderate change*** considering that one of the Condition to file as a Minor change was not met (Condition 3).

For the transfer of the pharmacopoeial assays, three groups suggested that it should be filed as a Minor change while one group suggested that it should be filed as a Moderate change. According to Change 51b [1], this would be true if the pharmacopoeial assay to be transferred was a potency assay (Condition 1). As the potency assay is a cell-based assay and that as of today, most if not all cell-based assays are considered non-pharmacopoeial, this imply that the potency test to be transferred is not a pharmacopoeial assay and the transfer should be reported as a ***Minor change***.

All groups considered that the supporting data for both sites were acceptable as comparative test data were available in support of the technical transfer of the non-pharmacopoeial assays and verification data were available for the transfer of the pharmacopoeial assay. A copy of a Certificate of GMP compliance by the regional regulatory authority was available as evidence of GMP compliance.

### 4.2 Case study 2: Change to the cell banks

#### 4.2.1 Case study 2-1: Change to the working cell banks (WCBs)

These changes fall under Appendix 2, Changes 4 and 5 [1].

**Table:** 

Description of Change	Conditions to be Fulfilled	Supporting Data	Reporting Categories
4. **Change in the cell bank testing/storage site**	5, 7	9	Minor

##### 4.2.1.1 Reporting categories

In order to file a change in cell bank testing/storage site as a Minor change, no changes should have been made to the tests/acceptance criteria used for the release of the cell bank (Condition 5). In addition, no changes should have been made to the storage conditions used for the cell bank and the transport conditions of the cell bank should have been validated (Condition 7).

Although Condition 7 is met, the MAH proposed to update the previously approved qualification protocol by replacing the Cell Line Authenticity test method. This is considered a change in the tests/acceptance criteria used for the release of the cell bank. Therefore, as Condition 5 is not met, the proposed change should be reported as a ***Moderate change*** and would require the filing of a PAS.

##### 4.2.1.2 Supporting data

For Change 4, Supporting data 9 requires the filing of evidence that the company/facility is GMP compliant [1]. One group considered that the manufacturer was lacking evidence of GMP compliance in the absence of GMP certificate of compliance while the other groups considered that the confirmation was acceptable. It was clarified in the workshop that not all NRAs issue a GMP compliance certificate after their inspection even if the site received a GMP compliance rating. Therefore, a confirmation from the MAH that the site has already been inspected by a competent regulatory authority and has received a GMP compliance status is normally sufficient as evidence of GMP compliance.

##### 4.2.1.3 Key discussion points

Questions were raised about the type of supporting data to be provided with the Moderate change as they are not defined for this category. It was clarified that generally, in such instances, the same supporting data as for the Minor change should be provided or judgement should be used to decide whether additional supporting should be provided. In this example, the change is considered to be at the next higher reporting category because changes have been made to the qualification protocol. Therefore, an updated copy of the qualification protocol would be considered appropriate to be filed with the ***Moderate change*** along with a demonstration that the new cell line authenticity test is suitable for its purpose.

**Table:** 

Description of Change	Conditions to be Fulfilled	Supporting Data	Reporting Categories
5. **Change in the cell bank qualification protocol**	None	3, 4	Moderate
	6	4	Minor

##### 4.2.1.4 Reporting categories

In order to file a change in the cell bank qualification protocol as a Minor change, the change should make the protocol more stringent (i.e. addition of new tests or narrowing of acceptance criteria, Condition 6).

As the replacement of the cell line authenticity test is not considered as being more stringent (no test added or narrowing of acceptance criteria), the proposed change is not deemed to meet the conditions required to file as a Minor change. Therefore, the change should be reported as a ***Moderate change*** and would require the filing of a PAS with Supporting data 3–4.

##### 4.2.1.5 Supporting data

Supporting data 3–4 require the MAHs to submit a justification of the change to the cell bank qualification protocol and an updated cell bank qualification protocol. As the MAH clarified that the change was made to the qualification protocol due to the limited commercial availability of the isoenzyme analysis test and greater sensitivity of the DNA fingerprinting-based assay and considering that an updated WCB qualification protocol was also included, the supporting data were deemed acceptable in support of this ***Moderate change***.

##### 4.2.1.6 Key discussion points

All groups classified this change as a ***Moderate change*** considering that Condition 6 was not met. However, one group highlighted the fact that the change in the method for a new identity test may be an improvement to the protocol if the new qualification method was considered more specific and sensitive compared to the previous test. It was suggested that method stringency could be determined by a risk assessment. It was reiterated that this is exactly the risk-based principles established in these guidelines. It is up to the MAH to provide a rationale to demonstrate how they meet Condition 6. If the rationale is accepted by the NRA, the change could be filed as Minor change. If not, the change should be file as a Moderate change. A risk assessment could have been provided by the MAH to support this position.

Finally, regarding the question if the two changes could be filed together, all groups agreed that as the two changes are related (i.e. involve the WCB), they may be filed in the same PAS, but the manufacture could want to file them separately. This was acknowledged.

#### 4.2.2 Case study 2-2: Change to the master cell banks (MCBs)

To address the questions related to changes to the MCBs, reference should be made to Appendix 2, Change 2 [1].

**Table:** 

Description of change	Conditions to be fulfilled	Supporting data	Reporting category
2. **Change to the cell banks:**			
Note: New cell substrates that are unrelated to the licensed master cell bank (MCB) or pre-MCB material may require a new application for marketing authorization or licence application.			
b. Generation of a new MCB	1	1, 2, 5–8	Moderate
**Conditions**			
1. The new MCB is generated from the original clone or from a pre-approved MCB and is grown in the same culture medium.			

##### 4.2.2.1 Reporting categories

According to this table, the reporting categories for the following scenarios would be:

• If the new MCB was generated from the same expression construct with same or closely related cell line (e.g. from CHO–K1 to CHO-DG44): a ***Major change*** as Condition 1 will not be met.
• If the new MCB was generated from a different expression construct with the same coding sequence and the same cell line: a ***Major change*** as Condition 1 will not be met.
• If the new MCB was generated from a different cell substrate (e.g. from CHO hamster cell line to NS0 murine cell line). It will likely require a ***New application for marketing authorization*** or license application as the new cell substrate is unrelated to the licensed master cell bank. As such, the glycosylation profile will likely be different (which could impact the PK/PD profile & efficacy of the product) and the difference in host cell proteins could also impact the immunogenicity profile of the product (which could lead to a different safety profile). This change in protein characteristics will lead to a different product, thus supporting the filing of a new application for marketing authorization.

##### 4.2.2.2 Key discussion points

All groups classified the three scenarios adequately. One group suggested that the generation of a new MCB from the same expression construct with same or closely related cell line (e.g. from CHO–K1 to CHO-DG44) could be filed as Major change if culture medium was different or as a Moderate change if the same culture medium was used. For this scenario, in order to classify the change as a Moderate change, Condition 1 requires that the new MCB be generated from the original clone or from a pre-approved MCB ***and*** be grown in the same culture medium. Therefore, as the new MCB proposed in Scenario 1 was not generated from the original clone or from a pre-approved MCB, Condition 1 is not met, and the change should be classified as a Major change.

### 4.3 Case study 3: Change to the fermentation process or cell culture

The introduction of Animal Component-Free (ACF) media to the Drug Substance (DS) manufacturing process is considered a change to the fermentation or cell culture process. It falls under Appendix 2, Change 6 [1].

**Table:** 

Description of Change	Conditions to be Fulfilled	Supporting Data	Reporting Categories
6. **Change to the fermentation or cell culture process:**			
a. A critical change (a change with high potential have an impact on the quality of the drug substance or drug product, e.g. incorporation of disposable bioreactor technology)	None	1-7, 9, 11	Major
b. A change with moderate potential to have an impact on the quality of the drug substance or drug product (e.g. extension of the in vitro cell age beyond validated parameters)	1, 3	1-6, 8, 10	Moderate
c. A noncritical change with minimal potential to have an impact on the quality of the drug substance or drug product, such as:	1-5, 7-10	1, 2, 4, 8	Minor
• a change in harvesting and/or pooling procedures which does not affect the method of manufacture, recovery, intermediate storage conditions, sensitivity of detection of adventitious agents or production scale;			
• duplication of a fermentation train; or			
• addition of similar/comparable bioreactors			

#### 4.3.1 Reporting categories

In order to determine whether it is considered a critical change, a change with moderate potential to have an impact on the quality of the drug substance or a non-critical change, the examples provided in the table can be used to help base the decision.

In this scenario, there is no change to the cell line and no change to the bioreactor. The change in cell culture media from animal component-containing media to animal component-free media may have an impact on the cell growth or on the glycosylation profile of the proteins. The relative impact depends on which ingredient(s) is/are impacted and this is not mentioned in the scenario. For example, if the change was to remove the Foetal Bovine Serum (FBS) from the cell culture media, this could have a major impact on the cell growth, protein expression and glycosylation. In this situation, it would be more appropriate to classify the change as a ***Major Change*** under Change 6a. Alternatively, if the change was to replace a non-critical component of the cell culture powder, the impact on the protein could be less and similar to an extension of the in-vitro cell age beyond validated parameters. In this situation, the change could be considered to have a moderate potential to impact the quality of the drug substance and could be filed as ***Moderate change*** under Change 6b. In any cases, the listed Conditions to be fulfilled should be used to help classifying the change.

#### 4.3.2 Supporting data

The supporting data depends on the relative potential to have an impact on the drug substance or drug product. The main difference between a Major and Moderate change is that for a Major change, three drug substance batches are required to support the change, compared to only one for a Moderate change. The number of batches to be placed on stability also varies accordingly. In this example, the sponsor provided 3 months of real-time data when 6 months is usually required. Therefore, it was recommended that a rationale should be requested as to why 3 months of real-time data is considered acceptable. Otherwise, more stability data should be provided. In addition, a commitment to notify the NRA of any failures should also be requested as it was missing from the original supporting data.

#### 4.3.3 Key discussion points

All group agreed that the introduction of Animal Component-Free (ACF) media to the drug substance manufacturing process may have an impact on the quality, safety or efficacy of the final product and as such, the change cannot be submitted as a Minor Change (Condition 9 not met).

At this point, the decision to file as a Major or Moderate change depends if Conditions 1 and 3 are fulfilled. As the scenario specifies that for the conversion to animal-free medium, there is no risk of introduction new viruses and that the impurity profile of the drug substance remains within the approved limits, three groups suggested that Conditions 1 and 3 would be fulfilled and the change could be filed as a ***Moderate change***. One group suggested that as the change could have a high potential to have an impact on the quality of the drug substance or drug product (e.g. glycosylation), it should be filed as a ***Major change***.

From a risk management point of view, both proposals are valid, and this was the intent of this case study. The description lacks details about the component that was replaced to make the media animal-free. As mentioned earlier, the removal of FBS from the media could have a significant impact on the cell growth and protein characteristics and would require the filing of a ***Major change***. Alternatively, a change in source from animal-derived to synthetic for a minor component of the cell culture medium could have a moderate potential to have an impact on the quality of the drug substance and the filing of a ***Moderate change*** would be considered appropriate. The key point of this case study is that when not enough details are available to classify a change, clarification should be requested from the MAH in order to make a proper risk-based decision.

### 4.4 Case study 4: Change in reference standards

To address the questions related to changes to the MCBs, reference should be made to Appendix 2, Change 23, 25 and 26 for drug substance or to Appendix 3, Changes 56, 58 and 59 for drug product [1].

For Drug substance:

**Table:** 

Description of change	Conditions to be fulfilled	Supporting data	Reporting category
23. **Replacement of a primary reference standard**	None	1, 2	Moderate
25. **Change of the reference standard from in-house (no relationship with international standard) to pharmacopoeial or international standard**	3	1, 2	Minor
26. **Qualification of a new batch of reference standard against the approved reference standard (**including **qualification of a new batch of a secondary reference standard against the approved primary standard)**	1	1, 2	Minor
**Conditions**			
1. Qualification of the new reference standard is in accordance with an approved protocol.			
2. The extension of the shelf-life of the reference standard is in accordance with an approved protocol.			
3. The reference standard is used for a physicochemical test.			

For Drug product:

**Table:** 

Description of change	Conditions to be fulfilled	Supporting data	Reporting category
56. **Replacement of a primary reference standard**	None	1, 2	Moderate
58. **Change of the reference standard from in-house (no relationship with international standard) to a pharmacopoeial or international standard**	3	1, 2	Minor
59. **Qualification of a new batch of reference standard against the approved reference standard (including qualification of a new batch of a secondary reference standard against the approved primary standard)**	1	2	Minor
**Conditions**			
1. The qualification of a new standard is carried out in accordance with an approved protocol.			
2. The extension of the shelf-life of the reference standard is carried out in accordance with an approved protocol.			
3. The reference standard is used for a physicochemical test.			

#### 4.4.1 Reporting categories

According to this table, the reporting categories for the following scenarios would be:

1. If the new reference standard was generated from the approved primary reference standard according to the approved protocol and is used for a physicochemical test at release, a ***Minor change*** according to Change 26 (and Change 59), as Condition 1 would be met. Whether the test is for a physicochemical test or a biological assay is not a factor to be considered for this type of change.
2. If the new reference standard was generated from an unapproved primary reference standard, a ***Moderate change*** according to Change 23 (and Change 56) as this would be considered a replacement of a primary reference standard.
3. If the new reference standard was qualified against the approved reference standard and was used as a control sample for an in-process control test, a ***Quality change with no impact*** as only the changes to reference standards used in the release of the drug substance or drug product should be reported. These changes are considered non-reportable.
4. If the new reference standard used for the potency assay test at release was changed from in-house to an international standard, a ***Moderate change*** according to Change 25 (and Change 58) as Condition 3 would not be met.
5. If a new lot of reference standard was qualified against the approved reference standard according to a modified qualification protocol (the MAH removed one of the purity test), a ***Moderate change*** according to Change 26 (and Change 59) as Condition 1 would not be met.
6. If a new lot of reference standard was qualified against the approved reference standard according to a modified qualification protocol (the MAH tightened the acceptance criteria for one of the purity test), it could be considered acceptable to report this change as a ***Minor change*** according to Change 26 (and Change 59) even if the approved protocol has been modified. As the change only pertains to the tightening of acceptance criteria and no tests have been deleted, this would not increase the risk associated with the change or make the protocol less stringent. This is a good example where the NRA should use their judgment to assess the appropriate reporting category. On the other side, reporting this change as a ***Moderate change*** would not be contrary to the guidelines.

#### 4.4.2 Key discussion points

Several scenarios were developed in this case study to cover a wide range of potential changes involving reference standards. While most of the groups classified all the proposed changes adequately, some were misclassified. One of the principles of the Guidelines is that only changes to reference standards used in the release of the drug substance or drug product should be reported. Changes to reference standards used as a control sample for an in-process control test do not need to be reported and are considered quality change with no impact. Another principle related to reference standards is that no changes should be reported as a Major change. According to Appendix 2, Changes 23–26 and Appendix 3, Changes, 56–59, the highest reporting category for a change to reference standard is a ***Moderate change***. Finally, due to the complexity of the potency assays and bioassays, the potential risks associated with a change to the reference standard used to control one of these assays is higher than for a reference standard used for a physicochemical test as described under Condition 3. As such, the reporting category will be higher for a change involving a potency or a bioassay than for a physicochemical assay.

## 5 Lessons learnt during the case studies

The objectives of this workshop were to facilitate implementation of WHO Guidelines into regulatory and manufacturing practices; to share the experience and examples through case studies and to identify a need for further technical support among the audience.

Active participation in the case study exercises has assisted regulators and manufacturers to have better understanding of key principles in evaluating changes made to biotherapeutic products.

Regulators and manufacturers were asked to review the case study data focusing on the questions mentioned under Section 3 of this article. They were invited to consider principles outlined in the WHO guidelines on procedures and data requirement for changes to approved biotherapeutic products [1] and to apply them to the case study scenario.

Throughout the workshop, several lessons were learned that identified knowledge gaps and the need for future trainings of NRAs. In particular, it was clarified that Conditions provided in Appendix 2 and Appendix 3 of the Guidelines are defined to mitigate the potential risk associated with a change. If any of the conditions outlined for a given change are not fulfilled, the change is automatically considered to be at the next higher reporting category.

On the other hand, if any of the supporting data outlined for a given change are not provided, are different from what listed, or are not considered applicable, adequate scientific justification should be provided or clarification should be requested to the MAH. It was highlighted during the workshop that when supporting data are not provided or are missing, this does not trigger the reclassification of the change at the next higher reporting category. As the risk assessment is based on the Conditions to be fulfilled, the reclassification of the change is applicable for the Conditions only. When the supporting data are not acceptable or not submitted without adequate rationale, the NRA should consider requesting clarification or rejecting the change.

During the cases study exercise, the group learned that for some changes, decisions may require situational judgement with respect to change scenario and the perceived risk, the conditions to be fulfilled and the supporting data to be submitted. This is particularly the case when the supporting data are not defined for a change that is filed at the next higher reporting category. In some cases, the same supporting data would apply where in other cases, additional supporting data would be required (e.g. for a change in cell-bank qualification protocol).

Another case study where scientific judgment should have been used is for the qualification of a new lot of reference standard when minor changes (e.g. tightening of acceptance criteria) were made to the qualification protocol. Even if one of the conditions to file as a minor change is that no changes have been made to the qualification protocol, tightening of acceptance criteria with no deletion of other tests will not increase the risk associated with the change or make the protocol less stringent. As such, even if non-critical changes were made to the qualification protocol, the NRAs should accept this change as a minor change.

The participants also learned in another case study involving the introduction of the animal component-free media to the drug substance manufacturing process, that when not enough details are available to classify a change, clarification should be requested to the MAH in order to make a proper risk-based decision. In that particular example, not enough details were provided on the component that were removed or synthetically manufactured. As such, the changes could be filed as Major or Moderate change depending on the specific details of the change.

Finally, it was clarified that when a change is not cover in the Guidelines, the MAH is encouraged to use scientific judgment, leverage competent regulatory guidance, or to contact the NRA to determine the potential impact of the change on the quality, safety and efficacy in order to discuss the appropriate reporting category.

## 6 Conclusions

The WHO Guidelines aim to ‘provide guidance for NRAs and MAHs on the regulation of changes to the original marketing authorization dossier or product licence for an approved biotherapeutic product in terms of: (a) the procedures and criteria for the appropriate categorization and reporting of changes; and (b) the data required to enable NRAs to evaluate the potential impact of the change on the quality, safety, and efficacy of the product’ [1]. The case studies in this article were developed to help NRAs' practical use of the Guidelines as well as understanding how the Guidelines work, i.e. (a) conditions are defined to mitigate the risk. When not met, higher reporting category must be filed; and (b) when supporting data are missing or unclear, clarification should be requested to the MAH. In addition, it should be noted that situational and scientific judgement may be needed in certain instance, e.g. for changes not specifically described in the Guidelines. Participants agreed that implementation of these WHO Guidelines (i.e. on reporting categories and data requirements) would contribute to harmonization or regulatory convergence on the requirements for post-approval changes of biotherapeutics globally and result in a more efficient approach of life-cycle management, e.g. by facilitating work-sharing/reliance models in those regions.

## Disclaimer

The authors alone are responsible for the views expressed in this article and they do not necessarily represent the views, decisions or policies of the institutions with which they are affiliated.

## Declaration of competing interest

The authors have disclosed no potential conflicts of interests.
